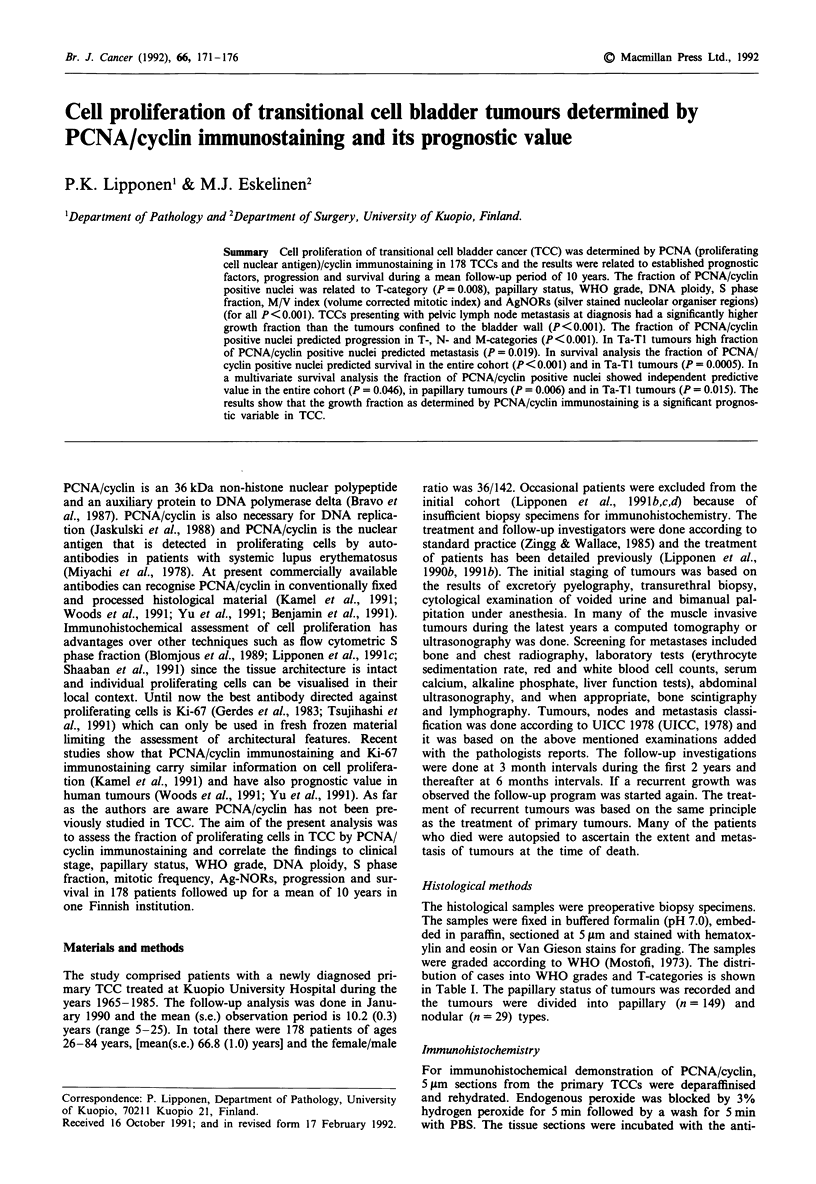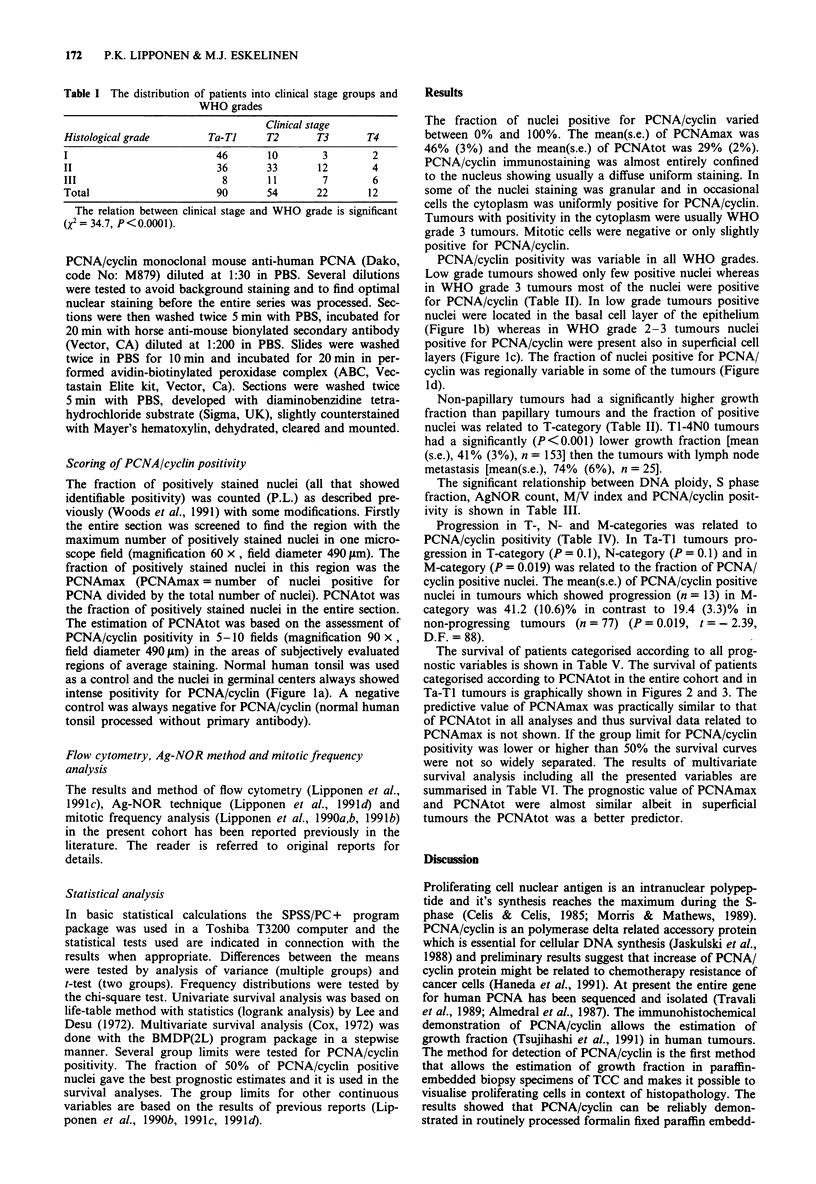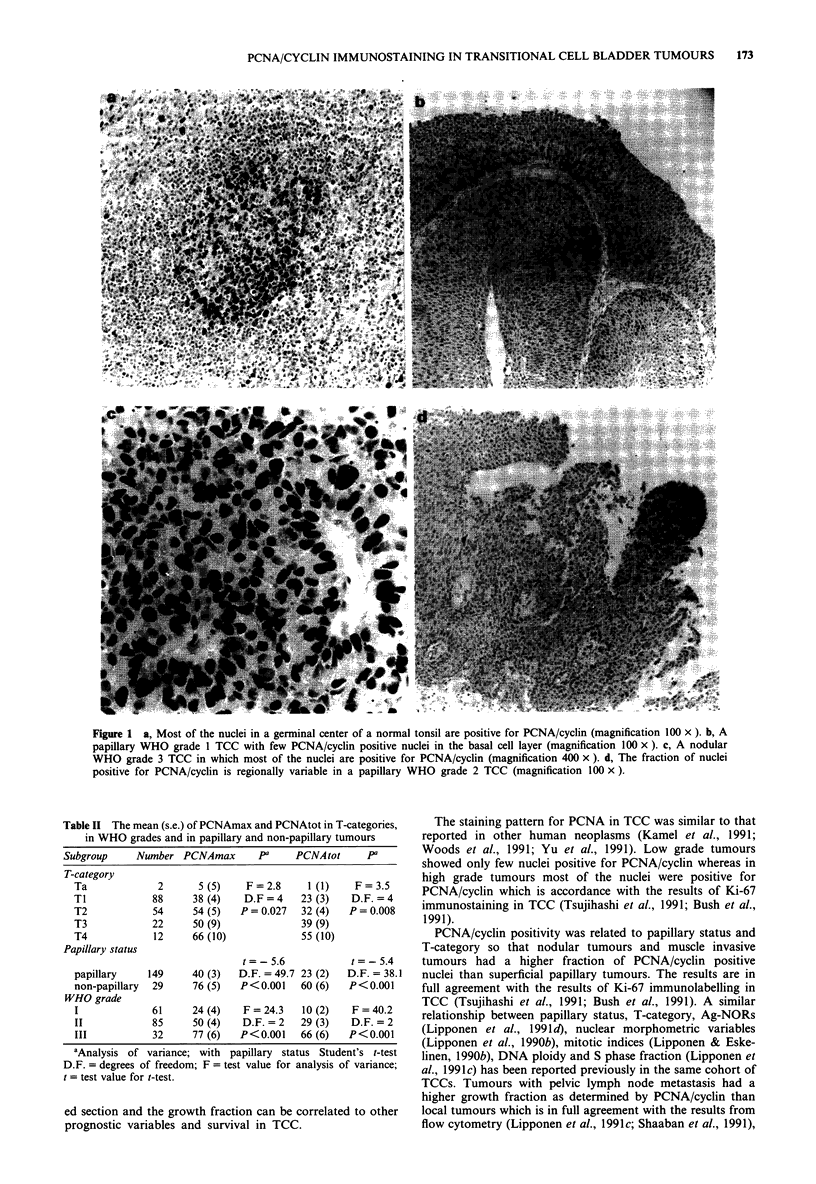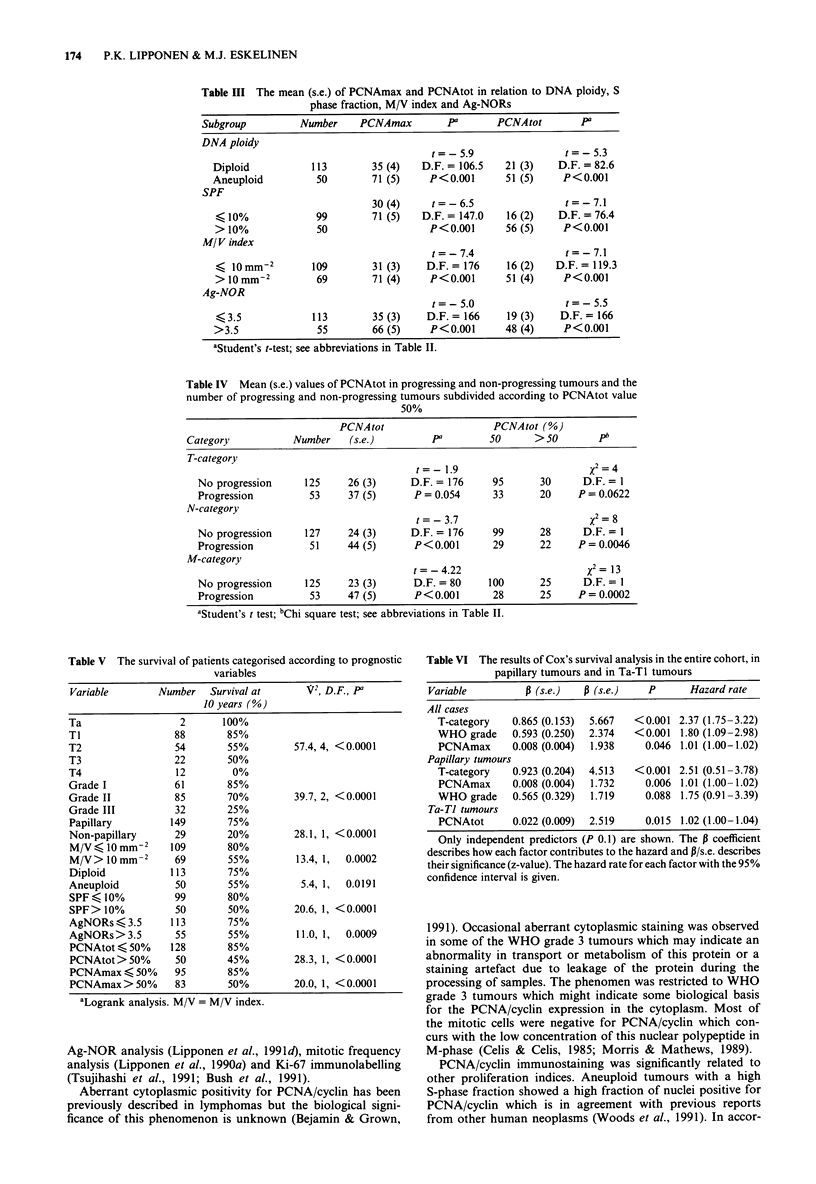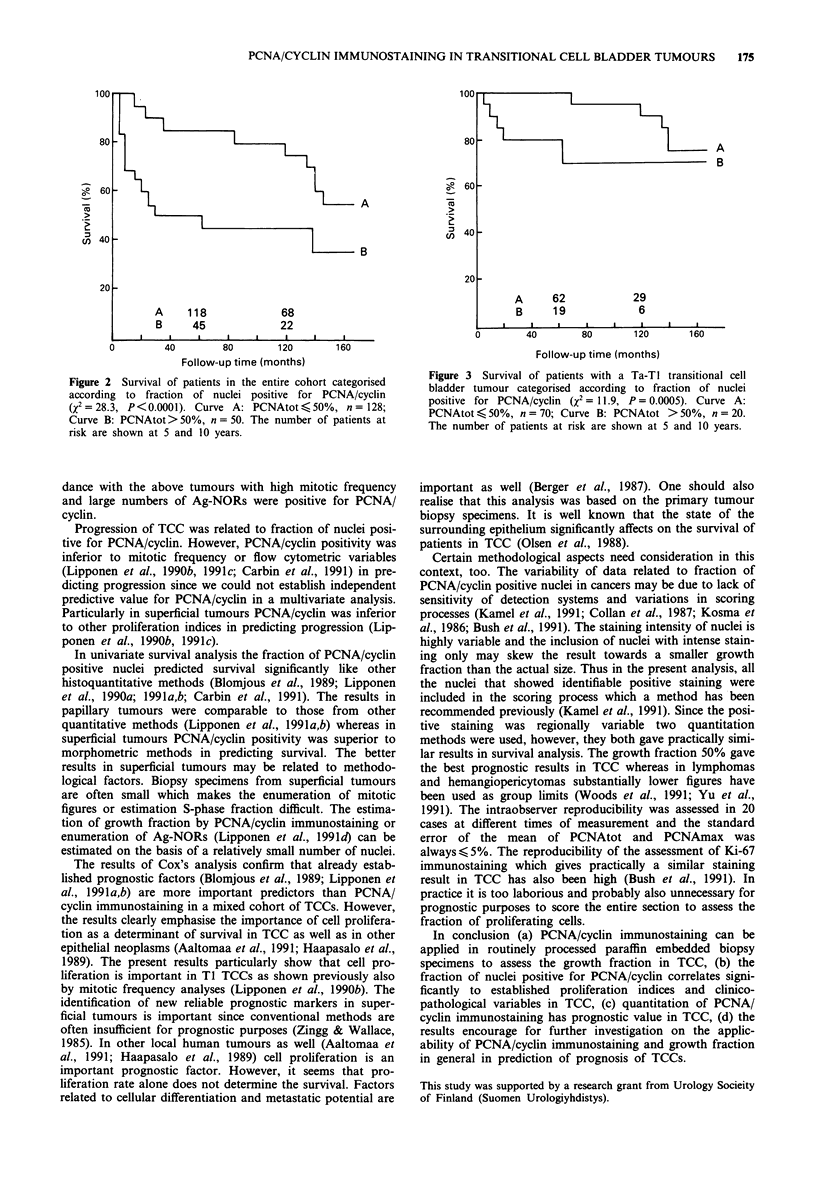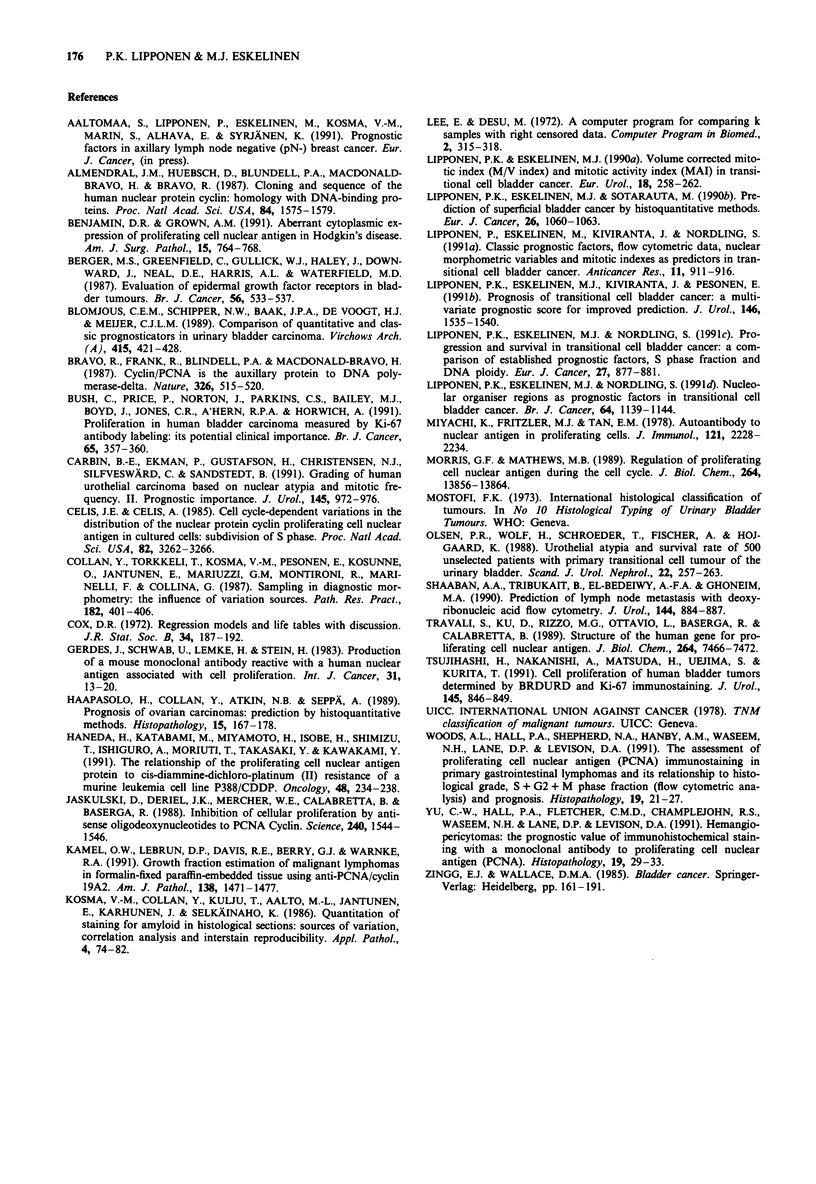# Cell proliferation of transitional cell bladder tumours determined by PCNA/cyclin immunostaining and its prognostic value.

**DOI:** 10.1038/bjc.1992.237

**Published:** 1992-07

**Authors:** P. K. Lipponen, M. J. Eskelinen

**Affiliations:** Department of Pathology, University of Kuopio, Finland.

## Abstract

**Images:**


Br. J. Cancer (1992), 66, 171–176 © Macmillan Press Ltd., 1992

### Cell proliferation of transitional cell bladder tumours determined by PCNA/cyclin immunostaining and its prognostic value

P.K. Lipponen[1] & M.J. Eskelinen[2]

[1]*Department of Pathology and* [2]*Department of Surgery, University of Kuopio, Finland.*

**Summary** Cell proliferation of transitional cell bladder cancer (TCC) was determined by PCNA (proliferating cell nuclear antigen)/cyclin immunostaining in 178 TCCs and the results were related to established prognostic factors, progression and survival during a mean follow-up period of 10 years. The fraction of PCNA/cyclin positive nuclei was related to T-category ($P = 0.008$), papillary status, WHO grade, DNA ploidy, S phase fraction, M/V index (volume corrected mitotic index) and AgNORs (silver stained nucleolar organiser regions) (for all $P < 0.001$). TCCs presenting with pelvic lymph node metastasis at diagnosis had a significantly higher growth fraction than the tumours confined to the bladder wall ($P < 0.001$). The fraction of PCNA/cyclin positive nuclei predicted progression in T-, N- and M-categories ($P < 0.001$). In Ta-T1 tumours high fraction of PCNA/cyclin positive nuclei predicted metastasis ($P = 0.019$). In survival analysis the fraction of PCNA/cyclin positive nuclei predicted survival in the entire cohort ($P < 0.001$) and in Ta-T1 tumours ($P = 0.0005$). In a multivariate survival analysis the fraction of PCNA/cyclin positive nuclei showed independent predictive value in the entire cohort ($P = 0.046$), in papillary tumours ($P = 0.006$) and in Ta-T1 tumours ($P = 0.015$). The results show that the growth fraction as determined by PCNA/cyclin immunostaining is a significant prognostic variable in TCC.

PCNA/cyclin is an 36 kDa non-histone nuclear polypeptide and an auxiliary protein to DNA polymerase delta (Bravo *et al.*, 1987). PCNA/cyclin is also necessary for DNA replication (Jaskulski *et al.*, 1988) and PCNA/cyclin is the nuclear antigen that is detected in proliferating cells by autoantibodies in patients with systemic lupus erythematosus (Miyachi *et al.*, 1978). At present commercially available antibodies can recognise PCNA/cyclin in conventionally fixed and processed histological material (Kamel *et al.*, 1991; Woods *et al.*, 1991; Yu *et al.*, 1991; Benjamin *et al.*, 1991). Immunohistochemical assessment of cell proliferation has advantages over other techniques such as flow cytometric S phase fraction (Blomjous *et al.*, 1989; Lipponen *et al.*, 1991*c*; Shaaban *et al.*, 1991) since the tissue architecture is intact and individual proliferating cells can be visualised in their local context. Until now the best antibody directed against proliferating cells is Ki-67 (Gerdes *et al.*, 1983; Tsujihashi *et al.*, 1991) which can only be used in fresh frozen material limiting the assessment of architectural features. Recent studies show that PCNA/cyclin immunostaining and Ki-67 immunostaining carry similar information on cell proliferation (Kamel *et al.*, 1991) and have also prognostic value in human tumours (Woods *et al.*, 1991; Yu *et al.*, 1991). As far as the authors are aware PCNA/cyclin has not been previously studied in TCC. The aim of the present analysis was to assess the fraction of proliferating cells in TCC by PCNA/cyclin immunostaining and correlate the findings to clinical stage, papillary status, WHO grade, DNA ploidy, S phase fraction, mitotic frequency, Ag-NORs, progression and survival in 178 patients followed up for a mean of 10 years in one Finnish institution.

### Materials and methods

The study comprised patients with a newly diagnosed primary TCC treated at Kuopio University Hospital during the years 1965–1985. The follow-up analysis was done in January 1990 and the mean (s.e.) observation period is 10.2 (0.3) years (range 5–25). In total there were 178 patients of ages 26–84 years, [mean(s.e.) 66.8 (1.0) years] and the female/male ratio was 36/142. Occasional patients were excluded from the initial cohort (Lipponen *et al.*, 1991*b,c,d*) because of insufficient biopsy specimens for immunohistochemistry. The treatment and follow-up investigators were done according to standard practice (Zingg & Wallace, 1985) and the treatment of patients has been detailed previously (Lipponen *et al.*, 1990*b*, 1991*b*). The initial staging of tumours was based on the results of excretory pyelography, transurethral biopsy, cytological examination of voided urine and bimanual palpitation under anesthesia. In many of the muscle invasive tumours during the latest years a computed tomography or ultrasonography was done. Screening for metastases included bone and chest radiography, laboratory tests (erythrocyte sedimentation rate, red and white blood cell counts, serum calcium, alkaline phosphate, liver function tests), abdominal ultrasonography, and when appropriate, bone scintigraphy and lymphography. Tumours, nodes and metastasis classification was done according to UICC 1978 (UICC, 1978) and it was based on the above mentioned examinations added with the pathologists reports. The follow-up investigations were done at 3 month intervals during the first 2 years and thereafter at 6 months intervals. If a recurrent growth was observed the follow-up program was started again. The treatment of recurrent tumours was based on the same principle as the treatment of primary tumours. Many of the patients who died were autopsied to ascertain the extent and metastasis of tumours at the time of death.

### *Histological methods*

The histological samples were preoperative biopsy specimens. The samples were fixed in buffered formalin (pH 7.0), embedded in paraffin, sectioned at 5 µm and stained with hematoxylin and eosin or Van Gieson stains for grading. The samples were graded according to WHO (Mostofi, 1973). The distribution of cases into WHO grades and T-categories is shown in Table I. The papillary status of tumours was recorded and the tumours were divided into papillary ($n = 149$) and nodular ($n = 29$) types.

### *Immunohistochemistry*

For immunohistochemical demonstration of PCNA/cyclin, 5 µm sections from the primary TCCs were deparaffinised and rehydrated. Endogenous peroxide was blocked by 3% hydrogen peroxide for 5 min followed by a wash for 5 min with PBS. The tissue sections were incubated with the anti-

Correspondence: P. Lipponen, Department of Pathology, University of Kuopio, 70211 Kuopio 21, Finland.
Received 16 October 1991; and in revised form 17 February 1992.

**Table I** The distribution of patients into clinical stage groups and WHO grades

| | Clinical stage | | | |
|---|---|---|---|---|
| *Histological grade* | *Ta-T1* | *T2* | *T3* | *T4* |
| I | 46 | 10 | 3 | 2 |
| II | 36 | 33 | 12 | 4 |
| III | 8 | 11 | 7 | 6 |
| Total | 90 | 54 | 22 | 12 |

The relation between clinical stage and WHO grade is significant ($\chi^2 = 34.7$, $P<0.0001$).

PCNA/cyclin monoclonal mouse anti-human PCNA (Dako, code No: M879) diluted at 1:30 in PBS. Several dilutions were tested to avoid background staining and to find optimal nuclear staining before the entire series was processed. Sections were then washed twice 5 min with PBS, incubated for 20 min with horse anti-mouse bionylated secondary antibody (Vector, CA) diluted at 1:200 in PBS. Slides were washed twice in PBS for 10 min and incubated for 20 min in performed avidin-biotinylated peroxidase complex (ABC, Vectastain Elite kit, Vector, Ca). Sections were washed twice 5 min with PBS, developed with diaminobenzidine tetrahydrochloride substrate (Sigma, UK), slightly counterstained with Mayer's hematoxylin, dehydrated, cleared and mounted.

### *Scoring of PCNA/cyclin positivity*

The fraction of positively stained nuclei (all that showed identifiable positivity) was counted (P.L.) as described previously (Woods *et al.*, 1991) with some modifications. Firstly the entire section was screened to find the region with the maximum number of positively stained nuclei in one microscope field (magnification 60 ×, field diameter 490 μm). The fraction of positively stained nuclei in this region was the PCNAmax (PCNAmax = number of nuclei positive for PCNA divided by the total number of nuclei). PCNAtot was the fraction of positively stained nuclei in the entire section. The estimation of PCNAtot was based on the assessment of PCNA/cyclin positivity in 5–10 fields (magnification 90 ×, field diameter 490 μm) in the areas of subjectively evaluated regions of average staining. Normal human tonsil was used as a control and the nuclei in germinal centers always showed intense positivity for PCNA/cyclin (Figure 1a). A negative control was always negative for PCNA/cyclin (normal human tonsil processed without primary antibody).

### *Flow cytometry, Ag-NOR method and mitotic frequency analysis*

The results and method of flow cytometry (Lipponen *et al.*, 1991*c*), Ag-NOR technique (Lipponen *et al.*, 1991*d*) and mitotic frequency analysis (Lipponen *et al.*, 1990*a,b*, 1991*b*) in the present cohort has been reported previously in the literature. The reader is referred to original reports for details.

### *Statistical analysis*

In basic statistical calculations the SPSS/PC+ program package was used in a Toshiba T3200 computer and the statistical tests used are indicated in connection with the results when appropriate. Differences between the means were tested by analysis of variance (multiple groups) and *t*-test (two groups). Frequency distributions were tested by the chi-square test. Univariate survival analysis was based on life-table method with statistics (logrank analysis) by Lee and Desu (1972). Multivariate survival analysis (Cox, 1972) was done with the BMDP(2L) program package in a stepwise manner. Several group limits were tested for PCNA/cyclin positivity. The fraction of 50% of PCNA/cyclin positive nuclei gave the best prognostic estimates and it is used in the survival analyses. The group limits for other continuous variables are based on the results of previous reports (Lipponen *et al.*, 1990*b*, 1991*c*, 1991*d*).

### Results

The fraction of nuclei positive for PCNA/cyclin varied between 0% and 100%. The mean(s.e.) of PCNAmax was 46% (3%) and the mean(s.e.) of PCNAtot was 29% (2%). PCNA/cyclin immunostaining was almost entirely confined to the nucleus showing usually a diffuse uniform staining. In some of the nuclei staining was granular and in occasional cells the cytoplasm was uniformly positive for PCNA/cyclin. Tumours with positivity in the cytoplasm were usually WHO grade 3 tumours. Mitotic cells were negative or only slightly positive for PCNA/cyclin.

PCNA/cyclin positivity was variable in all WHO grades. Low grade tumours showed only few positive nuclei whereas in WHO grade 3 tumours most of the nuclei were positive for PCNA/cyclin (Table II). In low grade tumours positive nuclei were located in the basal cell layer of the epithelium (Figure 1b) whereas in WHO grade 2–3 tumours nuclei positive for PCNA/cyclin were present also in superficial cell layers (Figure 1c). The fraction of nuclei positive for PCNA/cyclin was regionally variable in some of the tumours (Figure 1d).

Non-papillary tumours had a significantly higher growth fraction than papillary tumours and the fraction of positive nuclei was related to T-category (Table II). T1-4N0 tumours had a significantly ($P<0.001$) lower growth fraction [mean (s.e.), 41% (3%), $n = 153$] then the tumours with lymph node metastasis [mean(s.e.), 74% (6%), $n = 25$].

The significant relationship between DNA ploidy, S phase fraction, AgNOR count, M/V index and PCNA/cyclin positivity is shown in Table III.

Progression in T-, N- and M-categories was related to PCNA/cyclin positivity (Table IV). In Ta-T1 tumours progression in T-category ($P = 0.1$), N-category ($P = 0.1$) and in M-category ($P = 0.019$) was related to the fraction of PCNA/cyclin positive nuclei. The mean(s.e.) of PCNA/cyclin positive nuclei in tumours which showed progression ($n = 13$) in M-category was 41.2 (10.6)% in contrast to 19.4 (3.3)% in non-progressing tumours ($n = 77$) ($P = 0.019$, $t = -2.39$, D.F. = 88).

The survival of patients categorised according to all prognostic variables is shown in Table V. The survival of patients categorised according to PCNAtot in the entire cohort and in Ta-T1 tumours is graphically shown in Figures 2 and 3. The predictive value of PCNAmax was practically similar to that of PCNAtot in all analyses and thus survival data related to PCNAmax is not shown. If the group limit for PCNA/cyclin positivity was lower or higher than 50% the survival curves were not so widely separated. The results of multivariate survival analysis including all the presented variables are summarised in Table VI. The prognostic value of PCNAmax and PCNAtot were almost similar albeit in superficial tumours the PCNAtot was a better predictor.

### Discussion

Proliferating cell nuclear antigen is an intranuclear polypeptide and it's synthesis reaches the maximum during the S-phase (Celis & Celis, 1985; Morris & Mathews, 1989). PCNA/cyclin is an polymerase delta related accessory protein which is essential for cellular DNA synthesis (Jaskulski *et al.*, 1988) and preliminary results suggest that increase of PCNA/cyclin protein might be related to chemotherapy resistance of cancer cells (Haneda *et al.*, 1991). At present the entire gene for human PCNA has been sequenced and isolated (Travali *et al.*, 1989; Almedral *et al.*, 1987). The immunohistochemical demonstration of PCNA/cyclin allows the estimation of growth fraction (Tsujihashi *et al.*, 1991) in human tumours. The method for detection of PCNA/cyclin is the first method that allows the estimation of growth fraction in paraffin-embedded biopsy specimens of TCC and makes it possible to visualise proliferating cells in context of histopathology. The results showed that PCNA/cyclin can be reliably demonstrated in routinely processed formalin fixed paraffin embedd-

**Figure 1** **a**, Most of the nuclei in a germinal center of a normal tonsil are positive for PCNA/cyclin (magnification 100 ×). **b**, A papillary WHO grade 1 TCC with few PCNA/cyclin positive nuclei in the basal cell layer (magnification 100 ×). **c**, A nodular WHO grade 3 TCC in which most of the nuclei are positive for PCNA/cyclin (magnification 400 ×). **d**, The fraction of nuclei positive for PCNA/cyclin is regionally variable in a papillary WHO grade 2 TCC (magnification 100 ×).

**Table II** The mean (s.e.) of PCNAmax and PCNAtot in T-categories, in WHO grades and in papillary and non-papillary tumours

| *Subgroup* | *Number* | *PCNAmax* | $P^a$ | *PCNAtot* | $P^a$ |
|---|---|---|---|---|---|
| *T-category* | | | | | |
| Ta | 2 | 5 (5) | F = 2.8 | 1 (1) | F = 3.5 |
| T1 | 88 | 38 (4) | D.F = 4 | 23 (3) | D.F. = 4 |
| T2 | 54 | 54 (5) | $P = 0.027$ | 32 (4) | $P = 0.008$ |
| T3 | 22 | 50 (9) | | 39 (9) | |
| T4 | 12 | 66 (10) | | 55 (10) | |
| *Papillary status* | | | | | |
| | | | $t = -5.6$ | | $t = -5.4$ |
| papillary | 149 | 40 (3) | D.F. = 49.7 | 23 (2) | D.F. = 38.1 |
| non-papillary | 29 | 76 (5) | $P < 0.001$ | 60 (6) | $P < 0.001$ |
| *WHO grade* | | | | | |
| I | 61 | 24 (4) | F = 24.3 | 10 (2) | F = 40.2 |
| II | 85 | 50 (4) | D.F. = 2 | 29 (3) | D.F. = 2 |
| III | 32 | 77 (6) | $P < 0.001$ | 66 (6) | $P < 0.001$ |

[a]Analysis of variance; with papillary status Student's *t*-test D.F. = degrees of freedom; F = test value for analysis of variance; *t* = test value for *t*-test.

ed section and the growth fraction can be correlated to other prognostic variables and survival in TCC.

The staining pattern for PCNA in TCC was similar to that reported in other human neoplasms (Kamel *et al.*, 1991; Woods *et al.*, 1991; Yu *et al.*, 1991). Low grade tumours showed only few nuclei positive for PCNA/cyclin whereas in high grade tumours most of the nuclei were positive for PCNA/cyclin which is accordance with the results of Ki-67 immunostaining in TCC (Tsujihashi *et al.*, 1991; Bush *et al.*, 1991).

PCNA/cyclin positivity was related to papillary status and T-category so that nodular tumours and muscle invasive tumours had a higher fraction of PCNA/cyclin positive nuclei than superficial papillary tumours. The results are in full agreement with the results of Ki-67 immunolabelling in TCC (Tsujihashi *et al.*, 1991; Bush *et al.*, 1991). A similar relationship between papillary status, T-category, Ag-NORs (Lipponen *et al.*, 1991*d*), nuclear morphometric variables (Lipponen *et al.*, 1990*b*), mitotic indices (Lipponen & Eskelinen, 1990*b*), DNA ploidy and S phase fraction (Lipponen *et al.*, 1991*c*) has been reported previously in the same cohort of TCCs. Tumours with pelvic lymph node metastasis had a higher growth fraction as determined by PCNA/cyclin than local tumours which is in full agreement with the results from flow cytometry (Lipponen *et al.*, 1991*c*; Shaaban *et al.*, 1991),

**Table III** The mean (s.e.) of PCNAmax and PCNAtot in relation to DNA ploidy, S phase fraction, M/V index and Ag-NORs

| *Subgroup* | *Number* | *PCNAmax* | *P*[a] | *PCNAtot* | *P*[a] |
|---|---|---|---|---|---|
| *DNA ploidy* | | | | | |
| | | | $t = -5.9$ | | $t = -5.3$ |
| Diploid | 113 | 35 (4) | D.F. = 106.5 | 21 (3) | D.F. = 82.6 |
| Aneuploid | 50 | 71 (5) | $P<0.001$ | 51 (5) | $P<0.001$ |
| *SPF* | | | | | |
| | | 30 (4) | $t = -6.5$ | | $t = -7.1$ |
| ≤10% | 99 | 71 (5) | D.F. = 147.0 | 16 (2) | D.F. = 76.4 |
| >10% | 50 | | $P<0.001$ | 56 (5) | $P<0.001$ |
| *M/V index* | | | | | |
| | | | $t = -7.4$ | | $t = -7.1$ |
| ≤ 10 mm$^{-2}$ | 109 | 31 (3) | D.F. = 176 | 16 (2) | D.F. = 119.3 |
| >10 mm$^{-2}$ | 69 | 71 (4) | $P<0.001$ | 51 (4) | $P<0.001$ |
| *Ag-NOR* | | | | | |
| | | | $t = -5.0$ | | $t = -5.5$ |
| ≤3.5 | 113 | 35 (3) | D.F. = 166 | 19 (3) | D.F. = 166 |
| >3.5 | 55 | 66 (5) | $P<0.001$ | 48 (4) | $P<0.001$ |

[a]Student's *t*-test; see abbreviations in Table II.

**Table IV** Mean (s.e.) values of PCNAtot in progressing and non-progressing tumours and the number of progressing and non-progressing tumours subdivided according to PCNAtot value 50%

| *Category* | *Number* | *PCNAtot (s.e.)* | *P*[a] | *PCNAtot (%) 50* | *>50* | *P*[b] |
|---|---|---|---|---|---|---|
| *T-category* | | | | | | |
| | | | $t = -1.9$ | | | $\chi^2 = 4$ |
| No progression | 125 | 26 (3) | D.F. = 176 | 95 | 30 | D.F. = 1 |
| Progression | 53 | 37 (5) | $P = 0.054$ | 33 | 20 | $P = 0.0622$ |
| *N-category* | | | | | | |
| | | | $t = -3.7$ | | | $\chi^2 = 8$ |
| No progression | 127 | 24 (3) | D.F. = 176 | 99 | 28 | D.F. = 1 |
| Progression | 51 | 44 (5) | $P<0.001$ | 29 | 22 | $P = 0.0046$ |
| *M-category* | | | | | | |
| | | | $t = -4.22$ | | | $\chi^2 = 13$ |
| No progression | 125 | 23 (3) | D.F. = 80 | 100 | 25 | D.F. = 1 |
| Progression | 53 | 47 (5) | $P<0.001$ | 28 | 25 | $P = 0.0002$ |

[a]Student's *t* test; [b]Chi square test; see abbreviations in Table II.

**Table V** The survival of patients categorised according to prognostic variables

| *Variable* | *Number* | *Survival at 10 years (%)* | *V*$^2$*, D.F., P*[a] |
|---|---|---|---|
| Ta | 2 | 100% | |
| T1 | 88 | 85% | |
| T2 | 54 | 55% | 57.4, 4, <0.0001 |
| T3 | 22 | 50% | |
| T4 | 12 | 0% | |
| Grade I | 61 | 85% | |
| Grade II | 85 | 70% | 39.7, 2, <0.0001 |
| Grade III | 32 | 25% | |
| Papillary | 149 | 75% | |
| Non-papillary | 29 | 20% | 28.1, 1, <0.0001 |
| M/V ≤ 10 mm$^{-2}$ | 109 | 80% | |
| M/V > 10 mm$^{-2}$ | 69 | 55% | 13.4, 1, 0.0002 |
| Diploid | 113 | 75% | |
| Aneuploid | 50 | 55% | 5.4, 1, 0.0191 |
| SPF ≤ 10% | 99 | 80% | |
| SPF > 10% | 50 | 50% | 20.6, 1, <0.0001 |
| AgNORs ≤ 3.5 | 113 | 75% | |
| AgNORs > 3.5 | 55 | 55% | 11.0, 1, 0.0009 |
| PCNAtot ≤ 50% | 128 | 85% | |
| PCNAtot > 50% | 50 | 45% | 28.3, 1, <0.0001 |
| PCNAmax ≤ 50% | 95 | 85% | |
| PCNAmax > 50% | 83 | 50% | 20.0, 1, <0.0001 |

[a]Logrank analysis. M/V = M/V index.

**Table VI** The results of Cox's survival analysis in the entire cohort, in papillary tumours and in Ta-T1 tumours

| *Variable* | *β (s.e.)* | *β (s.e.)* | *P* | *Hazard rate* |
|---|---|---|---|---|
| *All cases* | | | | |
| T-category | 0.865 (0.153) | 5.667 | <0.001 | 2.37 (1.75–3.22) |
| WHO grade | 0.593 (0.250) | 2.374 | <0.001 | 1.80 (1.09–2.98) |
| PCNAmax | 0.008 (0.004) | 1.938 | 0.046 | 1.01 (1.00–1.02) |
| *Papillary tumours* | | | | |
| T-category | 0.923 (0.204) | 4.513 | <0.001 | 2.51 (0.51–3.78) |
| PCNAmax | 0.008 (0.004) | 1.732 | 0.006 | 1.01 (1.00–1.02) |
| WHO grade | 0.565 (0.329) | 1.719 | 0.088 | 1.75 (0.91–3.39) |
| *Ta-T1 tumours* | | | | |
| PCNAtot | 0.022 (0.009) | 2.519 | 0.015 | 1.02 (1.00–1.04) |

Only independent predictors (*P* 0.1) are shown. The β coefficient describes how each factor contributes to the hazard and β/s.e. describes their significance (z-value). The hazard rate for each factor with the 95% confidence interval is given.

Ag-NOR analysis (Lipponen *et al.*, 1991*d*), mitotic frequency analysis (Lipponen *et al.*, 1990*a*) and Ki-67 immunolabelling (Tsujihashi *et al.*, 1991; Bush *et al.*, 1991).

Aberrant cytoplasmic positivity for PCNA/cyclin has been previously described in lymphomas but the biological significance of this phenomenon is unknown (Bejamin & Grown, 1991). Occasional aberrant cytoplasmic staining was observed in some of the WHO grade 3 tumours which may indicate an abnormality in transport or metabolism of this protein or a staining artefact due to leakage of the protein during the processing of samples. The phenomen was restricted to WHO grade 3 tumours which might indicate some biological basis for the PCNA/cyclin expression in the cytoplasm. Most of the mitotic cells were negative for PCNA/cyclin which concurs with the low concentration of this nuclear polypeptide in M-phase (Celis & Celis, 1985; Morris & Mathews, 1989).

PCNA/cyclin immunostaining was significantly related to other proliferation indices. Aneuploid tumours with a high S-phase fraction showed a high fraction of nuclei positive for PCNA/cyclin which is in agreement with previous reports from other human neoplasms (Woods *et al.*, 1991). In accor-

**Figure 2** Survival of patients in the entire cohort categorised according to fraction of nuclei positive for PCNA/cyclin ($\chi^2 = 28.3$, $P<0.0001$). Curve A: PCNAtot$\leqslant$50%, $n = 128$; Curve B: PCNAtot>50%, $n = 50$. The number of patients at risk are shown at 5 and 10 years.

**Figure 3** Survival of patients with a Ta-T1 transitional cell bladder tumour categorised according to fraction of nuclei positive for PCNA/cyclin ($\chi^2 = 11.9$, $P = 0.0005$). Curve A: PCNAtot$\leqslant$50%, $n = 70$; Curve B: PCNAtot >50%, $n = 20$. The number of patients at risk are shown at 5 and 10 years.

dance with the above tumours with high mitotic frequency and large numbers of Ag-NORs were positive for PCNA/cyclin.

Progression of TCC was related to fraction of nuclei positive for PCNA/cyclin. However, PCNA/cyclin positivity was inferior to mitotic frequency or flow cytometric variables (Lipponen *et al.*, 1990*b*, 1991*c*; Carbin *et al.*, 1991) in predicting progression since we could not establish independent predictive value for PCNA/cyclin in a multivariate analysis. Particularly in superficial tumours PCNA/cyclin was inferior to other proliferation indices in predicting progression (Lipponen *et al.*, 1990*b*, 1991*c*).

In univariate survival analysis the fraction of PCNA/cyclin positive nuclei predicted survival significantly like other histoquantitative methods (Blomjous *et al.*, 1989; Lipponen *et al.*, 1990*a*; 1991*a,b*; Carbin *et al.*, 1991). The results in papillary tumours were comparable to those from other quantitative methods (Lipponen *et al.*, 1991*a,b*) whereas in superficial tumours PCNA/cyclin positivity was superior to morphometric methods in predicting survival. The better results in superficial tumours may be related to methodological factors. Biopsy specimens from superficial tumours are often small which makes the enumeration of mitotic figures or estimation S-phase fraction difficult. The estimation of growth fraction by PCNA/cyclin immunostaining or enumeration of Ag-NORs (Lipponen *et al.*, 1991*d*) can be estimated on the basis of a relatively small number of nuclei.

The results of Cox's analysis confirm that already established prognostic factors (Blomjous *et al.*, 1989; Lipponen *et al.*, 1991*a,b*) are more important predictors than PCNA/cyclin immunostaining in a mixed cohort of TCCs. However, the results clearly emphasise the importance of cell proliferation as a determinant of survival in TCC as well as in other epithelial neoplasms (Aaltomaa *et al.*, 1991; Haapasalo *et al.*, 1989). The present results particularly show that cell proliferation is important in T1 TCCs as shown previously also by mitotic frequency analyses (Lipponen *et al.*, 1990*b*). The identification of new reliable prognostic markers in superficial tumours is important since conventional methods are often insufficient for prognostic purposes (Zingg & Wallace, 1985). In other local human tumours as well (Aaltomaa *et al.*, 1991; Haapasalo *et al.*, 1989) cell proliferation is an important prognostic factor. However, it seems that proliferation rate alone does not determine the survival. Factors related to cellular differentiation and metastatic potential are important as well (Berger *et al.*, 1987). One should also realise that this analysis was based on the primary tumour biopsy specimens. It is well known that the state of the surrounding epithelium significantly affects on the survival of patients in TCC (Olsen *et al.*, 1988).

Certain methodological aspects need consideration in this context, too. The variability of data related to fraction of PCNA/cyclin positive nuclei in cancers may be due to lack of sensitivity of detection systems and variations in scoring processes (Kamel *et al.*, 1991; Collan *et al.*, 1987; Kosma *et al.*, 1986; Bush *et al.*, 1991). The staining intensity of nuclei is highly variable and the inclusion of nuclei with intense staining only may skew the result towards a smaller growth fraction than the actual size. Thus in the present analysis, all the nuclei that showed identifiable positive staining were included in the scoring process which a method has been recommended previously (Kamel *et al.*, 1991). Since the positive staining was regionally variable two quantitation methods were used, however, they both gave practically similar results in survival analysis. The growth fraction 50% gave the best prognostic results in TCC whereas in lymphomas and hemangiopericytomas substantially lower figures have been used as group limits (Woods *et al.*, 1991; Yu *et al.*, 1991). The intraobserver reproducibility was assessed in 20 cases at different times of measurement and the standard error of the mean of PCNAtot and PCNAmax was always$\leqslant$5%. The reproducibility of the assessment of Ki-67 immunostaining which gives practically a similar staining result in TCC has also been high (Bush *et al.*, 1991). In practice it is too laborious and probably also unnecessary for prognostic purposes to score the entire section to assess the fraction of proliferating cells.

In conclusion (a) PCNA/cyclin immunostaining can be applied in routinely processed paraffin embedded biopsy specimens to assess the growth fraction in TCC, (b) the fraction of nuclei positive for PCNA/cyclin correlates significantly to established proliferation indices and clinicopathological variables in TCC, (c) quantitation of PCNA/cyclin immunostaining has prognostic value in TCC, (d) the results encourage for further investigation on the applicability of PCNA/cyclin immunostaining and growth fraction in general in prediction of prognosis of TCCs.

This study was supported by a research grant from Urology Socieity of Finland (Suomen Urologiyhdistys).

### References

AALTOMAA, S., LIPPONEN, P., ESKELINEN, M., KOSMA, V.-M., MARIN, S., ALHAVA, E. & SYRJÄNEN, K. (1991). Prognostic factors in axillary lymph node negative (pN-) breast cancer. *Eur. J. Cancer*, (in press).

ALMENDRAL, J.M., HUEBSCH, D., BLUNDELL, P.A., MACDONALD-BRAVO, H. & BRAVO, R. (1987). Cloning and sequence of the human nuclear protein cyclin: homology with DNA-binding proteins. *Proc. Natl Acad. Sci. USA*, **84**, 1575–1579.

BENJAMIN, D.R. & GROWN, A.M. (1991). Aberrant cytoplasmic expression of proliferating cell nuclear antigen in Hodgkin's disease. *Am. J. Surg. Pathol.*, **15**, 764–768.

BERGER, M.S., GREENFIELD, C., GULLICK, W.J., HALEY, J., DOWNWARD, J., NEAL, D.E., HARRIS, A.L. & WATERFIELD, M.D. (1987). Evaluation of epidermal growth factor receptors in bladder tumours. *Br. J. Cancer*, **56**, 533–537.

BLOMJOUS, C.E.M., SCHIPPER, N.W., BAAK, J.P.A., DE VOOGT, H.J. & MEIJER, C.J.L.M. (1989). Comparison of quantitative and classic prognosticators in urinary bladder carcinoma. *Virchows Arch. (A)*, **415**, 421–428.

BRAVO, R., FRANK, R., BLINDELL, P.A. & MACDONALD-BRAVO, H. (1987). Cyclin/PCNA is the auxillary protein to DNA polymerase-delta. *Nature*, **326**, 515–520.

BUSH, C., PRICE, P., NORTON, J., PARKINS, C.S., BAILEY, M.J., BOYD, J., JONES, C.R., A'HERN, R.P.A. & HORWICH, A. (1991). Proliferation in human bladder carcinoma measured by Ki-67 antibody labeling: its potential clinical importance. *Br. J. Cancer*, **65**, 357–360.

CARBIN, B.-E., EKMAN, P., GUSTAFSON, H., CHRISTENSEN, N.J., SILFVESWÄRD, C. & SANDSTEDT, B. (1991). Grading of human urothelial carcinoma based on nuclear atypia and mitotic frequency. II. Prognostic importance. *J. Urol.*, **145**, 972–976.

CELIS, J.E. & CELIS, A. (1985). Cell cycle-dependent variations in the distribution of the nuclear protein cyclin proliferating cell nuclear antigen in cultured cells: subdivision of S phase. *Proc. Natl Acad. Sci. USA*, **82**, 3262–3266.

COLLAN, Y., TORKKELI, T., KOSMA, V.-M., PESONEN, E., KOSUNNE, O., JANTUNEN, E., MARIUZZI, G.M, MONTIRONI, R., MARINELLI, F. & COLLINA, G. (1987). Sampling in diagnostic morphometry: the influence of variation sources. *Path. Res. Pract.*, **182**, 401–406.

COX, D.R. (1972). Regression models and life tables with discussion. *J.R. Stat. Soc. B*, **34**, 187–192.

GERDES, J., SCHWAB, U., LEMKE, H. & STEIN, H. (1983). Production of a mouse monoclonal antibody reactive with a human nuclear antigen associated with cell proliferation. *Int. J. Cancer*, **31**, 13–20.

HAAPASOLO, H., COLLAN, Y., ATKIN, N.B. & SEPPÄ, A. (1989). Prognosis of ovarian carcinomas: prediction by histoquantitative methods. *Histopathology*, **15**, 167–178.

HANEDA, H., KATABAMI, M., MIYAMOTO, H., ISOBE, H., SHIMIZU, T., ISHIGURO, A., MORIUTI, T., TAKASAKI, Y. & KAWAKAMI, Y. (1991). The relationship of the proliferating cell nuclear antigen protein to cis-diammine-dichloro-platinum (II) resistance of a murine leukemia cell line P388/CDDP. *Oncology*, **48**, 234–238.

JASKULSKI, D., DERIEL, J.K., MERCHER, W.E., CALABRETTA, B. & BASERGA, R. (1988). Inhibition of cellular proliferation by antisense oligodeoxynucleotides to PCNA Cyclin. *Science*, **240**, 1544–1546.

KAMEL, O.W., LEBRUN, D.P., DAVIS, R.E., BERRY, G.J. & WARNKE, R.A. (1991). Growth fraction estimation of malignant lymphomas in formalin-fixed paraffin-embedded tissue using anti-PCNA/cyclin 19A2. *Am. J. Pathol.*, **138**, 1471–1477.

KOSMA, V.-M., COLLAN, Y., KULJU, T., AALTO, M.-L., JANTUNEN, E., KARHUNEN, J. & SELKÄINAHO, K. (1986). Quantitation of staining for amyloid in histological sections: sources of variation, correlation analysis and interstain reproducibility. *Appl. Pathol.*, **4**, 74–82.

LEE, E. & DESU, M. (1972). A computer program for comparing k samples with right censored data. *Computer Program in Biomed.*, **2**, 315–318.

LIPPONEN, P.K. & ESKELINEN, M.J. (1990*a*). Volume corrected mitotic index (M/V index) and mitotic activity index (MAI) in transitional cell bladder cancer. *Eur. Urol.*, **18**, 258–262.

LIPPONEN, P.K., ESKELINEN, M.J. & SOTARAUTA, M. (1990*b*). Prediction of superficial bladder cancer by histoquantitative methods. *Eur. J. Cancer*, **26**, 1060–1063.

LIPPONEN, P., ESKELINEN, M., KIVIRANTA, J. & NORDLING, S. (1991*a*). Classic prognostic factors, flow cytometric data, nuclear morphometric variables and mitotic indexes as predictors in transitional cell bladder cancer. *Anticancer Res.*, **11**, 911–916.

LIPPONEN, P.K., ESKELINEN, M.J., KIVIRANTA, J. & PESONEN, E. (1991*b*). Prognosis of transitional cell bladder cancer: a multivariate prognostic score for improved prediction. *J. Urol.*, **146**, 1535–1540.

LIPPONEN, P.K., ESKELINEN, M.J. & NORDLING, S. (1991*c*). Progression and survival in transitional cell bladder cancer: a comparison of established prognostic factors, S phase fraction and DNA ploidy. *Eur. J. Cancer*, **27**, 877–881.

LIPPONEN, P.K., ESKELINEN, M.J. & NORDLING, S. (1991*d*). Nucleolar organiser regions as prognostic factors in transitional cell bladder cancer. *Br. J. Cancer*, **64**, 1139–1144.

MIYACHI, K., FRITZLER, M.J. & TAN, E.M. (1978). Autoantibody to nuclear antigen in proliferating cells. *J. Immunol.*, **121**, 2228–2234.

MORRIS, G.F. & MATHEWS, M.B. (1989). Regulation of proliferating cell nuclear antigen during the cell cycle. *J. Biol. Chem.*, **264**, 13856–13864.

MOSTOFI, F.K. (1973). International histological classification of tumours. In *No 10 Histological Typing of Urinary Bladder Tumours*. WHO: Geneva.

OLSEN, P.R., WOLF, H., SCHROEDER, T., FISCHER, A. & HOJGAARD, K. (1988). Urothelial atypia and survival rate of 500 unselected patients with primary transitional cell tumour of the urinary bladder. *Scand. J. Urol. Nephrol.*, **22**, 257–263.

SHAABAN, A.A., TRIBUKAIT, B., EL-BEDEIWY, A.-F.A. & GHONEIM, M.A. (1990). Prediction of lymph node metastasis with deoxyribonucleic acid flow cytometry. *J. Urol.*, **144**, 884–887.

TRAVALI, S., KU, D., RIZZO, M.G., OTTAVIO, L., BASERGA, R. & CALABRETTA, B. (1989). Structure of the human gene for proliferating cell nuclear antigen. *J. Biol. Chem.*, **264**, 7466–7472.

TSUJIHASHI, H., NAKANISHI, A., MATSUDA, H., UEJIMA, S. & KURITA, T. (1991). Cell proliferation of human bladder tumors determined by BRDURD and Ki-67 immunostaining. *J. Urol.*, **145**, 846–849.

UICC. INTERNATIONAL UNION AGAINST CANCER (1978). *TNM classification of malignant tumours*. UICC: Geneva.

WOODS, A.L., HALL, P.A., SHEPHERD, N.A., HANBY, A.M., WASEEM, N.H., LANE, D.P. & LEVISON, D.A. (1991). The assessment of proliferating cell nuclear antigen (PCNA) immunostaining in primary gastrointestinal lymphomas and its relationship to histological grade, S + G2 + M phase fraction (flow cytometric analysis) and prognosis. *Histopathology*, **19**, 21–27.

YU, C.-W., HALL, P.A., FLETCHER, C.M.D., CHAMPLEJOHN, R.S., WASEEM, N.H. & LANE, D.P. & LEVISON, D.A. (1991). Hemangiopericytomas: the prognostic value of immunohistochemical staining with a monoclonal antibody to proliferating cell nuclear antigen (PCNA). *Histopathology*, **19**, 29–33.

ZINGG, E.J. & WALLACE, D.M.A. (1985). *Bladder cancer*. Springer-Verlag: Heidelberg, pp. 161–191.